# Characteristics of cardiovascular responses to an orthostatic challenge in trained spinal cord-injured individuals

**DOI:** 10.1186/s40101-018-0182-x

**Published:** 2018-09-29

**Authors:** Masahiro Itoh, Masako Yamaoka Endo, Tatsuya Hojo, Miho Yoshimura, Yoshiyuki Fukuoka

**Affiliations:** 1 Department of Physiology, Kumamoto University Graduate School of Life Sciences, 4-24-1, Kuhonji Chuou-ku, Kumamoto, 862-0976 Japan; 2 Department of Exercise Science and Physiology, Faculty of Human Culture and Science, Prefectural University of Hiroshima, Hiroshima, Japan; 3 Laboratory of Sports Medicine, Faculty of Health and Sport Science, Doshisha University, Kyoto, Japan; 4 Laboratory of Environmental Physiology, Faculty of Environmental and Symbiotic Sciences, Prefectural University of Kumamoto, Kumamoto, Japan; 5 Laboratory of Environmental Physiology, Faculty of Health and Sport Science, Doshisha University, Kyoto, Japan

**Keywords:** Orthostatic challenge, Spinal cord injury, Cardiovascular responses, Near-infrared spectroscopy

## Abstract

**Background:**

We investigated cardiovascular responses to an orthostatic challenge in trained spinal cord-injured (SCI) individuals compared to able-bodied (AB) individuals.

**Methods:**

A total of 23 subjects participated, divided into three groups: seven were trained as spinal cord-injured (Tr-SCI) individuals, seven were able-bodied individuals trained as runners (Tr-AB), and nine were untrained able-bodied individuals (UnTr-AB). We measured the cardiovascular autonomic responses in all three groups during each 5-min head-up tilt (HUT) of 0°, 40°, and 80°. Stroke volume (SV), heart rate (HR), and cardiac output (Qc) as cardiovascular responses were measured by impedance cardiography. Changes in deoxyhemoglobin (∆[HHb]) and total hemoglobin (∆[Hb_tot_]) concentrations of the right medial gastrocnemius muscle were measured using near-infrared spectroscopy (NIRS).

**Results:**

As the HUT increased from 0° to 80°, Tr-SCI group showed less change in SV at all HUT levels even if HR increased significantly. Mean arterial pressure (MAP) also did not significantly increase as tilting increased from 0° to 80°. Regarding peripheral vascular responses, the alterations of ∆[Hb_tot_] from 0° to 80° were less in Tr-SCI group compared to AB individuals.

**Conclusion:**

There is a specific mechanism whereby blood pressure is maintained during a HUT in Tr-SCI group with the elicitation of peripheral vasoconstriction and the atrophy of the vascular vessels in paraplegic lower limbs, which would be associated with less change in SV in response to an orthostatic challenge.

## Background

A spinal cord injury (SCI) leads to dramatic central and peripheral cardiovascular adaptation. Decreases in cardiac output (Qc) and dimension have been reported in individuals with SCIs [1, 2]. Below the level of SCI lesions, increased peripheral vascular resistance [3], reduced peripheral capillarization [4], and decreased conduit artery diameters have also been observed [5–7]. These findings indicate that less blood pooling may occur in paraplegic lower limbs. However, in individuals with a SCI, orthostatic hypotension resulting from an acute or progressive decline in blood pressure (BP) > 10–15 mmHg in an erect position during standing or tilting may result in poor tolerance for standing, thus prolonging the rehabilitation time and worsening the deleterious effects of remaining immobile [8]. The mechanism that underlies maintenance of BP must therefore play an important role in the tilt-induced increase in leg vascular tone in SCI patients corresponding to reduced central circulatory hypokinesis.

By contrast, despite the absence of central sympathetic control of limb vascular adjustment, SCI patients showed remarkable orthostatic tolerance during posture stress and their BP level was maintained during orthostatic challenges by augmented local vasoconstriction, most likely as part of the myogenic response [9]. Theisen et al. also showed a vasoconstriction response during leg dependency in paraplegia, suggesting that a veno-arteriolar axon reflex (VAR) or myogenic activity was present in spinal cord-injured individuals [10]. It is thus still unknown whether the blood pressure of individuals with SCIs can be maintained during an orthostatic challenge (as able-bodied healthy subjects can).

It has been demonstrated that individuals with aerobically fit paraplegia had significantly higher maximal cardiorespiratory fitness compared to individuals with sedentary lifestyle paraplegia [11]. During an incremental arm exercise, individuals with aerobically fit paraplegia developed a 34–44% Qc advantage and had a greater stroke volume (SV) relative to their inactive counterparts [12]. If wheelchair exercise training would induce greater Qc and mitigate reduced central circulatory hypokinesis, the arterial inflow of blood into paraplegic limbs might be augmented. In addition, some degree of resultant blood pooling could occur when the individual is in an upright position. However, it is not known whether trained SCI individuals can maintain their BP level in response to an orthostatic challenge. Near-infrared spectroscopy (NIRS) is a useful tool that can observe alterations in the microvascular blood flow into skeletal muscles when a subject is in a resting state. The present study comprehensively evaluated the interaction of central and peripheral circulation for BP maintenance in trained SCI individuals.

## Methods

### Participants and aerobic fitness

Seven trained SCI male individuals (Tr-SCI 34.1 ± 4.9 years old, body weight [BW] 58.9 ± 3.7 kg, VO2 max 27.3 ± 1.72 ml kg^−1^ min^−1^ during arm cranking, lesion level: thoracic vertebrae 6th (Th6) – lumbar vertebrae 1st (Lu1)) underwent a continuous wheelchair-basketball training program (basic skill and game training, approximately 2 h/day, 3 days/week) with an intensity of approximately 80% HR peak [1]. None of the Tr-SCI group took any antihypertensive agents. The thickness of the subcutaneous adipose tissue (ATT) in the medial gastrocnemius muscle region was determined by B-mode ultrasound (model Logiq 400; GE-Yokogawa Medical Systems, Tokyo) with the Tr-SCI group in the sitting position. All individuals had incomplete lesions classified as American Spinal Injury Association [ASIA] class B [13]. The physical characteristics of the Tr-SCI group, the amount of time since they incurred their SCIs, and their time spent practicing basketball are shown in Table 1.

**Table 1:** Physical characteristics and basketball career length for trained spinal cord-injured individuals

Subjects	Sex	Age (years)	Weight (kg)	Time since injury (months)	Length of basketball career (years)	Level of injury (spinal cord lesion)
a	Male	24	50	75	4.5	Th12 and Lu1
b	Male	27	52	84	5.0	Th12
c	Male	52	70	385	31.0	Lu1
d	Male	20	73	38	2.0	Th11
e	Male	51	51	319	24.0	Th11 and Th12
f	Male	37	64	128	9.0	Th7
g	Male	28	52	59	5.0	Th6
mean ± SE		34.1 ± 4.9	58.9 ± 3.7	155.4 ± 52.3	11.5 ± 4.3	

Seven healthy trained able-bodied male individuals (Tr-AB 19.4 ± 0.3 years old, 168.9 ± 1.8 cm, BW 56.6 ± 1.7 kg, VO2 max 60.9 ± 1.61 ml kg^−1^ min^−1^ during running) and nine healthy untrained able-bodied male individuals (UnTr-AB; 21.0 ± 0.4 years old, 170.5 ± 1.1 cm, BW 62.8 ± 2.1 kg, VO2 max no measurement) constituted the two other groups tested. The individuals in the Tr-AB group were university level long-distance runners who had run approximately 300 km month^−1^ each over the prior 10 years. The individuals in the UnTr-AB group were university students who did not engage in any regular sports activity. All Tr-AB and UnTr-AB group had no history of cardiovascular diseases and were healthy as indicated by medical history, physical examination, BP (< 140/90 mmHg), and 12-lead electrocardiogram (ECG). The experimental protocol was approved by the ethics committee of the Institutional Review Board of the Prefectural University of Kumamoto. All subjects provided written consent for their participation after they were fully informed about the study. The investigation was performed in compliance with the Declaration of Helsinki.

### Tilt table protocol

All three groups of individuals underwent experimental sessions (15 min) using a customized head-up tilt (HUT; Ishinuki steel Co., Kumamoto) table from 0° (supine position), to 40° (tilting position), to 80° (standing position) for 5 min. The body in the Tr-SCI group was fixed on three portions of chest, upper limbs, and lower limbs by three clinical belts with the strong hook-and-loop fastener from both sides of the tilt bed. The Tr-SCI group could stand the foot plate in their shoes. Their shoes produced friction on foot plate to prevent their slip. Tilt gradients were changed slowly to prevent ankle clonus in the Tr-SCI group. During three steps of HUT, we assumed that that energy expenditure in the individuals was almost constant. On each data collection day, individuals reported to the laboratory at least 2 h after their last meal. They were asked to avoid caffeine, alcohol ingestion, and strenuous exercise for 24 h before the test. The temperature and relative humidity of the laboratory were maintained at 25 °C and 50%, respectively.

### Measurements and data analysis

The central hemodynamic values of SV and Qc were continuously determined by an impedance method [14–16] using a computer-based automated technique with an impedance plethysmograph (AI-601G, Nihon Koden, Tokyo). Four disposable electrodes were placed on the neck and chest. The analog change in impedance (ΔZ) data and the voltage changes in the thorax were detected.

To avoid the effects of respiration and movement on impedance signals, we averaged the ΔZ values over three heartbeats, with the R-wave from the ECG serving as the trigger [17]. The Qc was estimated noninvasively by measuring the changes in electrical impedance (*Z*_0_) transthoracically. Changes in transthoracic impedance have an inverse relationship to changes in the volume of fluid in the thorax. The SV was calculated as follows: SV = *ρ* × (*L*^2^/*Z*_0_^2^) × ET × (dZ/dt_max_), where SV indicates the stroke volume; ρ, the resistivity of the conductor; *L*^2^/*Z*_0_^2^, the thoracic impedance (*Ω*), which is inversely proportional to the amount of fluid (ohms) in the thorax; ET, the ejection time; and dZ/dt_max_, the maximum deflection (Ω/s) of the dZ/dt waveform during the ET. Trained SCI patients had already the information of blood examination data before this experiment. The mean value of *Hct* was 38.5 ± 3.43 (SD) %. As the rho was calculated by Geddes and Sadler equation (rho = 53.2e^0.022Hct^), the averaged values in rho was 124.4 ± 9.4 Ω cm. Otherwise, the value of rho in both AB groups was constant of 135 Ω cm. Both AB groups did not collect blood for analysis of *Hct*.

The beat-by-beat heart rate (HR) was continuously monitored by ECG (AT-601G; Nihon Kohden, Tokyo), which was conducted using transistor-transistor logic signal intervals synchronized with the R-wave of the ECG by CM5 leads. All data were adopted using a data acquisition system (PowerLab system, A/D Instruments, Castle Hill, NSW, Australia) with an interval resolution of 2 ms (i.e., sampling interval at 500 Hz). A customized software program was used to identify a stable and noise-independent fiducial point on all R-waves for each recording.

Systolic and diastolic blood pressure (SBP, DBP) values were measured using a sphygmomanometer (DM-500, Muranaka, Osaka) at heart level on the upper right arm as it rested alongside the subject’s body. The BP was measured twice at 2 min and 4 min at each gradient. The total peripheral resistance (TPR) was calculated when the BP was available. The TPR measure was defined as the mean arterial pressure (MAP) per cardiac output in mmHg L^−1^ min^−1^. The MAP was calculated using DBP and SBP, using the formula MAP = DBP + 1/3 (SBP − DBP).

The oxygenation and deoxygenation profiles of the right medial gastrocnemius muscle were recorded during the HUT test using continuous-wave NIRS (BOM-L1 TR, Omega Wave, Tokyo). The system monitored the concentration in oxyhemoglobin (HbO_2_), deoxyhemoglobin (HHb), and total hemoglobin (Hb_tot_), which were calculated from the light attenuation change by using the modified Beer-Lambert law [18]. ∆[Hb_tot_] was the sum of ∆[HHb] and ∆[HbO_2_]. Pulsed light was emitted at 1-s intervals from the emission probe at four different wavelengths (775, 810, 850, and 910 nm) and was detected, as a function of distance, using a three-segment photodiode detection probe that received NIRS signals at 2 Hz. The distance between the emitter and receiver was 40 mm, and the penetration depth was approx. half of the distance between the emitter and the receiver, i.e., 20 mm. This has been validated in both healthy persons and patients [19] and patients [20, 21].

The NIRS data gathered represented the relative concentration changes in the hemoglobin chromophores and were therefore not representative of absolute tissue O_2_ values. As ∆[HbO_2_], ∆[HHb], and ∆[Hb_tot_] were measured as a change from the resting baseline values, the probe gain was zero set prior to testing with the subject at rest in a supine position (0°).

### Statistical analysis

All data are expressed as mean ± standard error (SE). Two-way analysis of variance (ANOVA) was performed to determine whether the central and peripheral parameters were significantly different during the three stages (0°, 40°, and 80°) among the three groups (Tr-SCI, Tr-AB, and UnTr-AB). If significant effects of tilt were observed, Fisher’s PLSD test was used to evaluate the differences between two trials (vs. 0 gradient degree in each group/vs. Tr-AB in each stage). A mixed effects analysis of variance (ANOVA) was used to separately to compare additional significant main (group and HUT) and interaction (group X HUT) effects of HR and BP to the HUT procedures. A probability (*p*) value of < 0.05 was regarded as significant.

## Results

There were statistically significant differences in age between the Tr-SCI and Tr-AB groups (*F*_(2,20)_ = 4.28, *p* < 0.01) and the Tr-SCI and UnTr-AB groups (*F*_(2,20)_ = 3.58, *p* < 0.01).

The ATT in the medial gastrocnemius muscle region averaged 8.7 ± 0.8 mm in the Tr-SCI group. Even though the ATT in the two present AB groups could not be measured, our recent ATT data in a different study averaged 3.0 ± 0.8 mm in runners and 3.9 ± 1.2 mm in untrained young subjects (unpubl. observations).

**Fig. 1:**
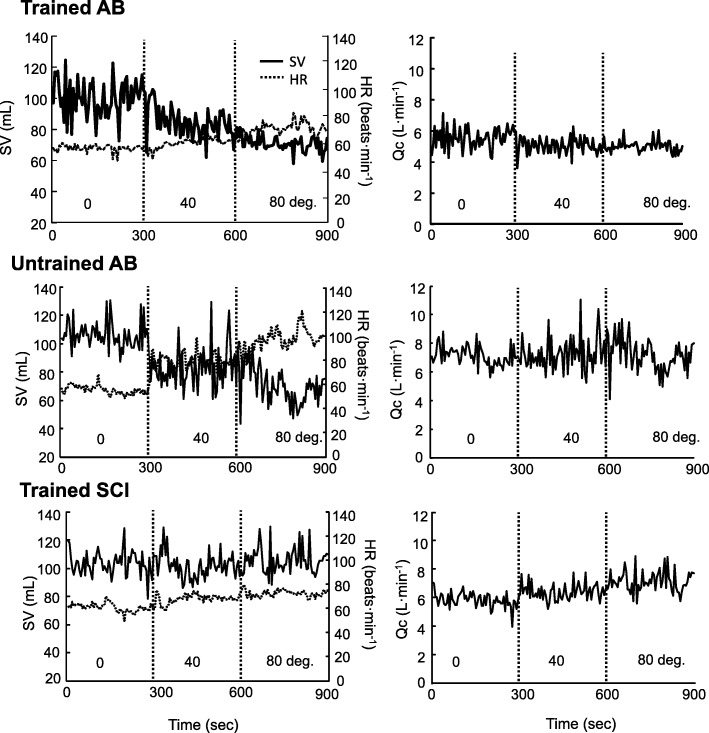
Time progression of stroke volume (SV), heart rate (HR), and cardiac output (Qc) in three representative individuals of trained (Tr-) spinal cord-injured (SCI) individuals and Tr-/untrained (UnTr-) able-bodied (AB) individuals

Figure 1 shows the central circulatory responses for HR, SV, and Qc during the HUT tests. Significantly increased HR and inversely decreased SV were proportionally observed in association with increased HUT from 0° to 80° in both AB groups, whereas the Tr-SCI group exhibited no change in SV at any degree of tilt.

**Fig. 2:**
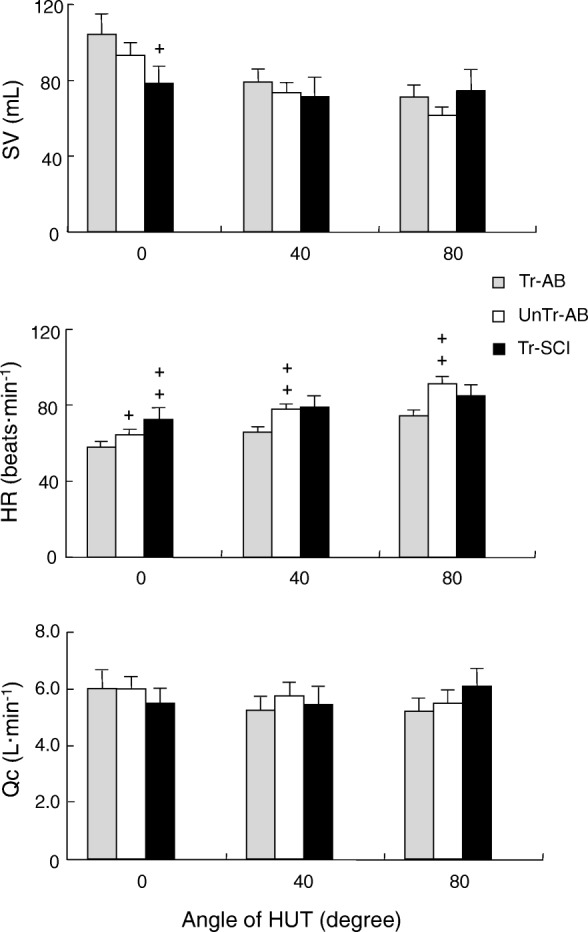
Changes in SV, HR, and Qc with the difference of gradient degree (0°, 40°, and 80°) for each group (light gray, Tr-AB; white, UnTr-AB; black, Tr-SCI). Data are mean ± SE. +, ++ *p* < 0.05, 0.01 vs. Tr-AB in each degree

In the supine position (0°), the HR in the Tr-SCI group (73 ± 6 bpm) was significantly greater than that in the Tr-AB group (58 ± 3 bpm, *F*_(2,20)_ = 3.63, *p* < 0.01), whereas a significantly lower SV at 0° was found in the Tr-SCI group (78 ± 9 mL) in comparison with the Tr-AB group (104 ± 11 mL, *F*_(2,20)_ = 4.10, *p* < 0.05) (Fig. 2). The HR in both the Tr-AB and UnTr-AB groups increased significantly with proportionally increased gradients (from 58 ± 3 bpm to 74 ± 3 bpm in Tr-AB; from 64 ± 2 bpm to 91 ± 4 bpm in UnTr-AB, *F* = 3.89, *F*_(2,20)_ = 3.63, *p* < 0.01, respectively) (Table 2). The HR in Tr-SCI group also increased significantly with proportionally increased gradients (from 73 ± 6 bpm to 85 ± 6 bpm, *F*_(2,20)_ = 4.10, *p* < 0.01, respectively) (Table 2).

**Table 2:** Vascular dynamic responses during three stages of the tilt table

	0° (rest)	40°	80°
Tr-AB			
HR (beats min^−1^)	58 ± 3	66 ± 3 **	74 ± 3 **
SV (mL min^−1^)	104 ± 11	79 ± 6 *	71 ± 6 *
Qc (L min^−1^)	6.0 ± 0.6	5.2 ± 0.5	5.2 ± 0.5
SBP (mmHg)	116 ± 2	117 ± 3	118 ± 4
DBP (mmHg)	61 ± 2	67 ± 3	69 ± 3
MAP (mmHg)	80 ± 3	83 ± 4	85 ± 3 *
TPR (mmHg L^−1^ min^−1^)	15 ± 3	17 ± 3	17 ± 2
UnTr-AB			
HR (beats min^−1^)	64 ± 2	78 ± 2 **	91 ± 4 **
SV (mL min^−1^)	93 ± 6	73 ± 5 **	62 ± 4 **
Qc (L min^−1^)	5.9 ± 0.4	5.7 ± 0.4	5.6 ± 0.4
SBP (mmHg)	101 ± 10	109 ± 7	113 ± 5
DBP (mmHg)	58 ± 6	67 ± 4 **	72 ± 2 **
MAP (mmHg)	76 ± 7	85 ± 5 **	90 ± 3 **
TPR (mmHg L^−1^ min^−1^)	14 ± 1	16 ± 2	17 ± 2
Tr-SCI			
HR (beats min^−1^)	73 ± 6	79 ± 6 **	85 ± 6 **
SV (mL min^−1^)	78 ± 9	71 ± 10	74 ± 11
Qc (L min^−1^)	5.5 ± 0.5	5.4 ± 0.6	6.1 ± 0.6
SBP (mmHg)	115 ± 4	118 ± 2	124 ± 4
DBP (mmHg)	70 ± 2	73 ± 1	76 ± 2
MAP (mmHg)	85 ± 3	88 ± 1	92 ± 2
TPR (mmHg L^−1^ min^−1^)	16 ± 2	17 ± 2	16 ± 2

Heart rate (HR), stroke volume (SV), cardiac output (Qc), systolic blood pressure (SBP), diastolic blood pressure (DBP), mean arterial pressure (MAP), and total peripheral resistance (TPR) were determined. Data are shown by mean ± SE

*,** *p* < 0.05, 0.01 vs. 0 degree in each group, respectively

The corresponding SV in both AB groups was significantly decreased at 80° compared to 0° (from 104 ± 11 mL to 71 ± 6 mL in Tr-AB; from 93 ± 6 mL to 62 ± 4 mL in UnTr-AB, *F*_(2,20)_ = 3.89, *F*_(2,20)_ = 3.63, *p* < 0.05, *p* < 0.01, respectively). On the other hand, the Tr-SCI group had a distinct profile in that the SV did not change significantly, even though the HR increased significantly, as the gradient of the tilt table increased (Table 2). The resultant Qc was not significantly different in any group or at any gradient of the tilt table.

Significantly increased MAP values at 80° in Tr-AB group and significantly increased DBP and MAP values were observed at both 40° and 80° in UnTr-AB group, whereas no significant differences in SBP, DBP, MAP, or TPR changes were observed in the Tr-SCI group as the gradient increased from 0° to 40° and 80° (Table 2). For all three groups at each gradient, there were no significant differences in any SBP variables.

**Fig. 3:**
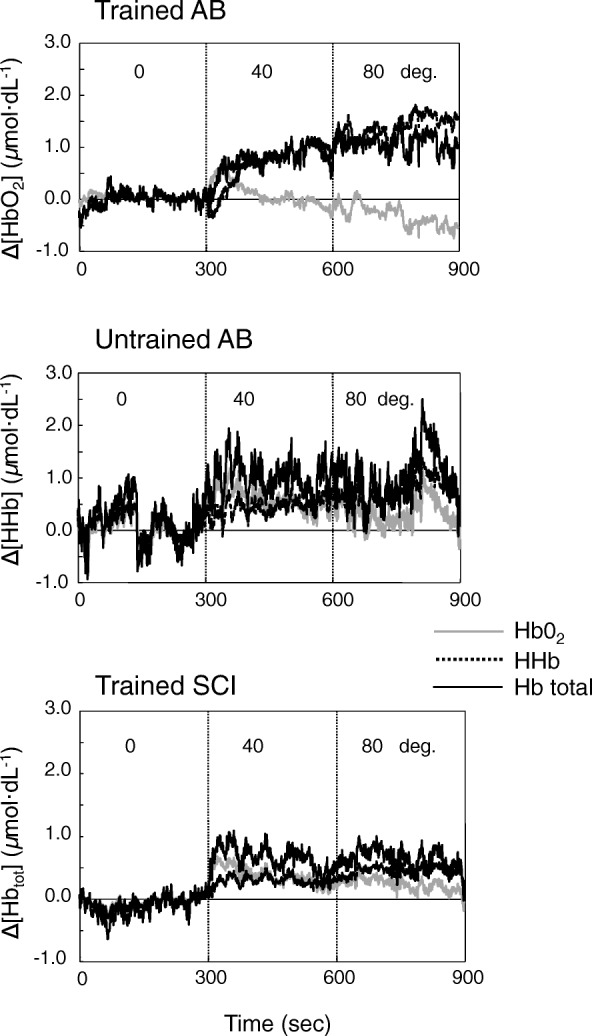
Time progression in peripheral circulatory responses estimated from ∆[HbO_2_] (gray), ∆[HHb] (dotted), and ∆[Hb_tot_] (solid) during an orthostatic challenge in four representative individuals of the Tr-SCI, Tr-AB, and UnTr-AB groups

**Fig. 4:**
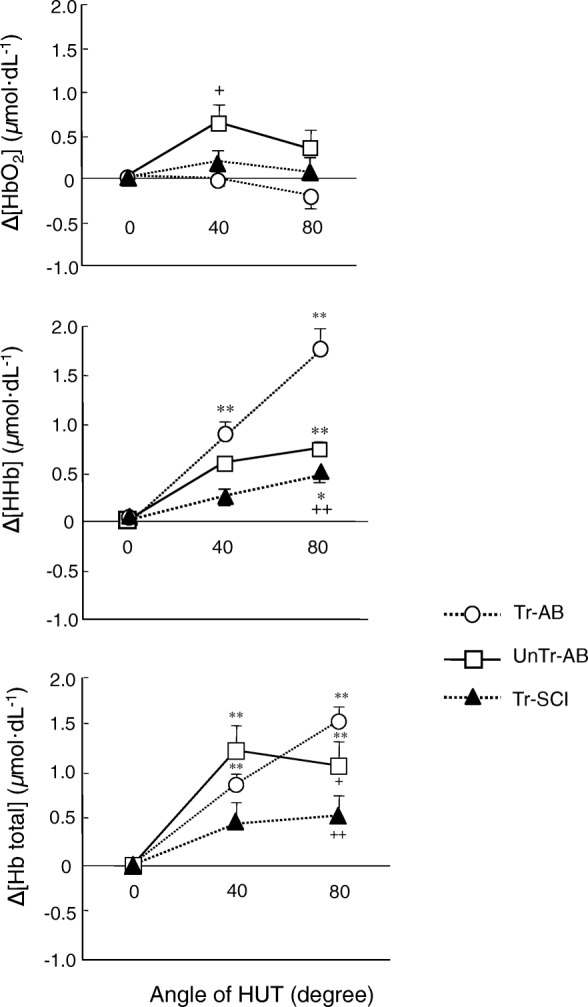
Changes in peripheral circulatory responses, ∆[HbO_2_], ∆[HHb], and ∆[Hb_tot_] with the difference of gradient degree (0°, 40°, and 80°) for each group (white circle: Tr-AB, white square: UnTr-AB, black trianle: Tr-SCI). Data are mean ± SE. *, ** *p* < 0.05, 0.01 vs. 0 gradient degree at each group. +, ++ *p* < 0.05, 0.01 vs. Tr-AB

Figure 3 shows the representative peripheral circulatory responses estimated from ∆[HbO_2_], ∆[HHb], and ∆[Hb_tot_] in each group during an orthostatic challenge. ∆[HbO_2_], ∆[HHb], and ∆[Hb_tot_] in the gastrocnemius muscle increased greatly as the gradient increased from 0° to 40° and 80° in UnTr-AB subjects. Interestingly, there was a significant difference in ∆[HbO_2_] between Tr-AB and UnTr-AB subjects at 40° (Tr-AB, − 0.01 ± 0.08; UnTr-AB, 0.62 ± 0.21 μmol dL^−1^, *F*_(2,20)_ = 3.49, *p* < 0.05, Fig. 4). However, the average ∆[HbO_2_] in the Tr-SCI subjects remained stable at all gradients.

In all three groups, there was a common trend in which the ∆[HHb] proportionally increased with the gradients, and a significant difference in ∆[HHb] was observed between 0° and 80° even in the Tr-SCI group (*F*_(2,20)_ = 3.55, *p* < 0.01, Fig. 4). In particular, the ∆[HHb] in the Tr-AB group (1.74 ± 0.23 μmol dL^−1^) increased compared to the Tr-SCI group at 80° (Tr-SCI, 0.45 ± 0.07 μmol dL^−1^, *F*_(2,20)_ = 3.49, *p* < 0.01) and compared to the UnTr-AB group (0.73 ± 0.08 μmol dL^−1^, *F*_(2,20)_ = 3.49, *p* < 0.05).

∆[Hb_tot_], which was the sum of ∆[HHb] and ∆[HbO_2_], was mostly reflected in the alterations in ∆[HHb] and was significantly increased as the tilt gradient increased (Fig. 4). At 80° HUT, the ∆[Hb_tot_] in the Tr-AB group (1.53 ± 0.16 μmol dL^−1^) was shown to be significantly greater than those in the UnTr-AB and Tr-SCI groups (1.05 ± 0.25, 0.52 ± 0.22, μmol dL^−1^, *F*_(2,20)_ = 3.40, *p* < 0.05, *p* < 0.01, respectively). Compared to the UnTr-AB group, significantly smaller ∆[Hb_tot_] values were observed in Tr-SCI group (*F*_(2,20)_ = 3.49, *p* < 0.05). Thus, the status of the change (AB vs. SCI) could be reflected by ∆[HHb] and ∆[HbO_2_] responses to orthostatic stress associated with central circulation.

## Discussion

The present study examined the differential changes in central and peripheral circulatory responses to an orthostatic challenge in the Tr-SCI group in comparison with the Tr-AB and UnTr-AB groups at different tilt gradients (0°, 40°, and 80°). Regarding the peripheral circulation corresponding to the central circulation, small alterations of ∆[HHb] and ∆[Hb_tot_] from 0° to 40° to 80° were shown by the Tr-SCI group. Significantly greater increases of ∆[HHb] and ∆[Hb_tot_] occurred in the Tr-AB group (*p* < 0.05), corresponding to significantly decreased SV and increased HR in both AB groups. Therefore, trained individuals with spinal cord injuries (Tr-SCI) are capable of maintaining their MAP even in response to an orthostatic challenge, and the mechanism underlying this maintenance of MAP may be similar to or the same as that by which peripheral vasoconstriction and less blood pooling are elicited in paraplegic limbs.

### Hemodynamic responses to an orthostatic challenge

In Tr-SCI group, the HR in a supine position was significantly higher than that in the two AB groups. It was reported previously that paraplegics had a higher HR in a supine position compared to able-bodied controls [22]. In another study, SCI individuals exhibited significantly higher resting values of plasma renin and catecholamines (particularly epinephrine) than able-bodied individuals [23]. Among higher HR responders in able-bodied individuals, the plasma norepinephrine responses were all greater than those exhibited by individuals with lower HR responses [24]. We therefore speculate that in the present Tr-SCI group, the cardiovascular effects of chronically elevated vasoactive hormones might play a very significant role in setting the mean HR (Fig. 2).

In both AB groups of the present study, significantly increased HR and inversely decreased SV were proportionally observed in association with increased HUT from 0° to 80°, whereas the Tr-SCI group exhibited less change in SV at all degrees of tilt. The resultant Qc showed no significant differences among any of the groups or gradient degrees. In addition, it has been suggested that in the Tr-SCI group, the venous return blood volume remained constant with less blood pooling under orthostatic stress. Sympathetic neurogenic vasoconstriction is quantitatively important in terms of successful responses to orthostasis [25]. Aslan et al. indicated that an SCI results in decreased stimulation of arterial baroreceptors and less engagement of feedback control [26].

However, in the present investigation, the Tr-SCI group was able to maintain their MAP during HUT tests even though their responses showed no significant changes, whereas in the AB groups, the MAP and DBP were significantly increased at increased gradient degrees. This suggests that the baroreflex sensitivity in the Tr-SCI group might not be activated because less venous blood pools from the limbs to the thoracic region [27]. Tr-SCI group was characterized by slightly but significantly increased ∆[HHb], even though the HR and the BP responses were not greatly altered. This might be due to the maintenance of residual sympathetically mediated vasoconstriction for paraplegic individuals with lesions below the Th6 level of the incomplete spinal cord.

### Peripheral vascular responses estimated using NIRS

At an 80° tilt, the ∆[HHb] in the Tr-AB group was increased approximately threefold compared to the Tr-SCI and compared to the UnTr-AB groups; the ∆[Hb_tot_] was mostly reflected in the alterations in ∆[HHb]. In previous studies involving SCI individuals, the central blood volume was found to be decreased by approximately 20% compared to healthy subjects at supine rest [28] because there is less blood pooling volume and/or fewer blood vessels in the lower limb muscles [29]. The former possibility would result from the increasing leg vascular resistance in individuals with an SCI during HUT in order to maintain the mean arterial pressure [30]. The latter possibility would result from the gastrocnemius circulation volume per se being reduced due to the atrophy of the vascular vessels [29].

The vascular atrophy of the peripheral vascular bed was confirmed by some studies that observed a smaller femoral artery (FA) or reduced capillary supply in SCI individuals compared to able-bodied subjects [3, 7]. Consistent with the reduced FA diameter, the FA blood flow in SCI individuals (from 220 to 150 mL min^−1^) during HUT also appeared to be reduced when compared with values reported in able-bodied individuals (from 350 to 230 mL min^−1^) [9]. Indeed, the increase in leg vascular resistance during HUT not only in SCI individuals but also in healthy controls was unaffected during an intra-arterial infusion of phentolamine (an adrenergic antagonist), irrespective of whether the sympathetic baroreflex was intact [31].

The Tr-SCI group was characterized by slightly but significantly increased ∆[HHb] at 80° HUT compared to 0°, demonstrating that even the trained SCI individuals also exhibited venous distensibility and venous capacity in the lower limbs [9]. In addition, differences in ∆[Hb_tot_] and ∆[HHb] between our Tr-AB and UnTr-AB individuals might be due to the increased number of vessels resulting from the Tr-AB subjects’ endurance exercise training. This is thought to be caused by the volume of blood pooling having increased much more in the Tr-AB group than in UnTr-AB group because endurance exercise training imparts a powerful stimulus for vascular remodeling [32].

### Study limitations

In general, the 20- and 30-year-old subjects would be defined as young individuals. The age difference between the trained SCI and two AB groups is not very important, because the metabolism in working muscles would be similar in potential between a 30-year-old and a 20-year-old [1].

The continuous-wave (CW)-NIRS we used in the present study assesses relative rather than absolute [HbO_2_], [HHb], and [Hb_tot_] values, which may have obscured the underlying response(s). The ATT in the medial gastrocnemius muscle region averaged 8.7 ± 0.8 mm in the Tr-SCI group, which might influence the optic strength of each wavelength in the Tr-SCI group. Thus, there may be a possibility that we underestimated the optical coefficients in the cases of > 5 mm ATT [33]. The ATT in young subjects were mostly < 5 mm, as observed in our other study [34], even though the ATT in the two present AB groups could not be measured.

By contrast, Binzoni et al. reported that the deoxygenation [HHb] of CW-NIRS did not vary as a function of the fat layer, whereas the oxygenation [HbO_2_] was definitely higher in muscle than in fat, as shown by the lower ATT data [35]. In the present study, as the larger alterations in ∆[HHb] and ∆[Hb_tot_] were observed during HUT, the clinical implications for CW-NIRS might have been less influenced.

Since we did not measure EMG in paralyzed limbs as muscle spasms were often assessed in previous studies [36], thus, we could not quantify muscle activity (e.g., spasm). This is one of the technical limitations in the present study; however, we carefully observed and did not find any symptoms of muscle spasm in all patients. Despite this, future studied should be warranted.

## Conclusions

In a supine posture, the Tr-SCI group had significantly higher HR and lower SV values compared to the two AB groups. During subsequent HUT tests, Tr-SCI group exhibited less decreased SV and small alterations of ∆[HHb] and ∆[Hb_tot_] compared to those in both AB groups. MAP also did not significantly increase as the tilting increased from 0° to 80°. These results suggest that there is less blood pooling due to peripheral vasoconstriction and fewer blood vessels in paraplegic lower limbs of exercise-trained SCI individuals. These results are associated with less change in SV in response to an orthostatic challenge.

## Abbreviations

∆[HbO_2_]: Oxyhemoglobin
∆[Hb_tot_]: Total hemoglobin
∆[HHb]: Deoxyhemoglobin
AB: Able-bodied individual
HR: Heart rate
HUT: Head-up tilt
MAP: Mean arterial pressure
Qc: Cardiac output
SBP & DBP: Systolic and diastolic blood pressures
SCI: Spinal cord-injured individual
SV: Stroke volume
TPR: Total peripheral resistance
Tr-, UnTr-: Trained-, untrained individuals
